# Recanalization by magnetic compression anastomosis for complete bile duct obstruction and retrieval of a migrated magnet

**DOI:** 10.1055/a-2134-7324

**Published:** 2023-08-21

**Authors:** Ikuhiro Kobori, Takuji Noro, Musashi Takada, Koichi Soga, Masaya Tamano, Hideyuki Yoshitomi, Eigoro Yamanouchi

**Affiliations:** 1 Department of Gastroenterology, Dokkyo Medical University Saitama Medical Center, Saitama, Japan; 2 Department of Surgery, Dokkyo Medical University Saitama Medical Center, Saitama, Japan; 3 Department of Radiology, International University of Health and Welfare Hospital, Tochigi, Japan

Complete obstruction of the bile duct after hepatic resection is rare and extremely difficult to treat. We report recanalization using magnetic compression anastomosis and retrieval of a magnet that migrated into the intrahepatic bile duct.

A 77-year-old man developed complete obstruction of the bile duct due to a biliary fistula following hepatic resection for hepatocellular carcinoma ( Fig. 1 ). Breakthrough of the bile duct stricture through the endoscopic retrograde cholangiopancreatography (ERCP) route was unsuccessful, and percutaneous transhepatic biliary drainage (PTBD) was performed. After attempts to break through the stricture via the PTBD route were also unsuccessful, magnetic compression anastomosis was performed ( Fig. 2 ).

**Fig. 1:**
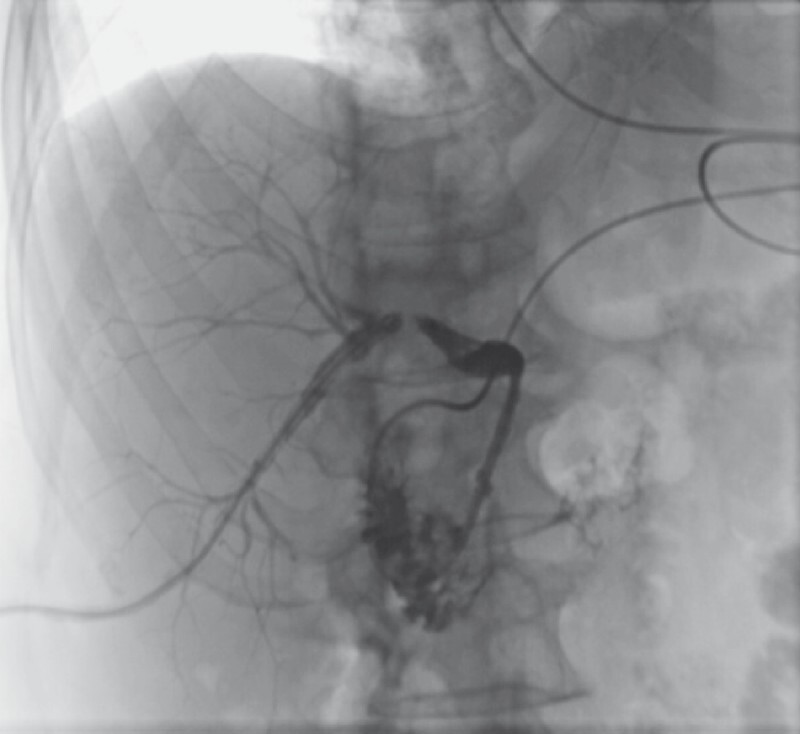
Fluoroscopic images show complete obstruction of the bile duct.

**Fig. 2:**
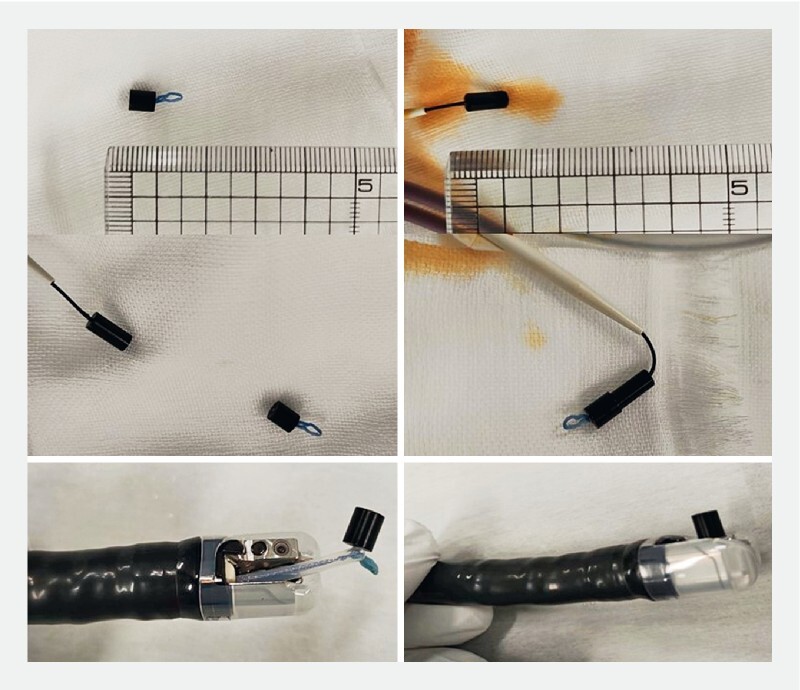
Photographs of the magnets.

A thick sheath was inserted into the PTBD route, and one magnet was inserted through the sheath to the site of bile duct obstruction ( Video 1 ). A 10-mm covered self-expandable metallic stent (cSEMS) was placed in the bile duct for insertion of the other magnet from the ERCP route into the bile duct. The magnet was carefully grasped by the snare of the ERCP scope, brought to the papilla, and inserted through the cSEMS into the bile duct obstruction. The locations of the two magnets were checked under fluoroscopy ( Fig. 3 ), and recanalization of the occlusion site was confirmed by injection of contrast medium 2 weeks later ( Fig. 4 ). However, the magnet then migrated into the intrahepatic bile duct side and was difficult to retrieve from the ERCP route. A cholangioscope was finally inserted into the PTBD route and the magnet was successfully retrieved ( Fig. 5 ).

**Video 1** Recanalization by magnetic compression anastomosis for complete bile duct obstruction and retrieval of the migrated magnet.


**Fig. 3:**
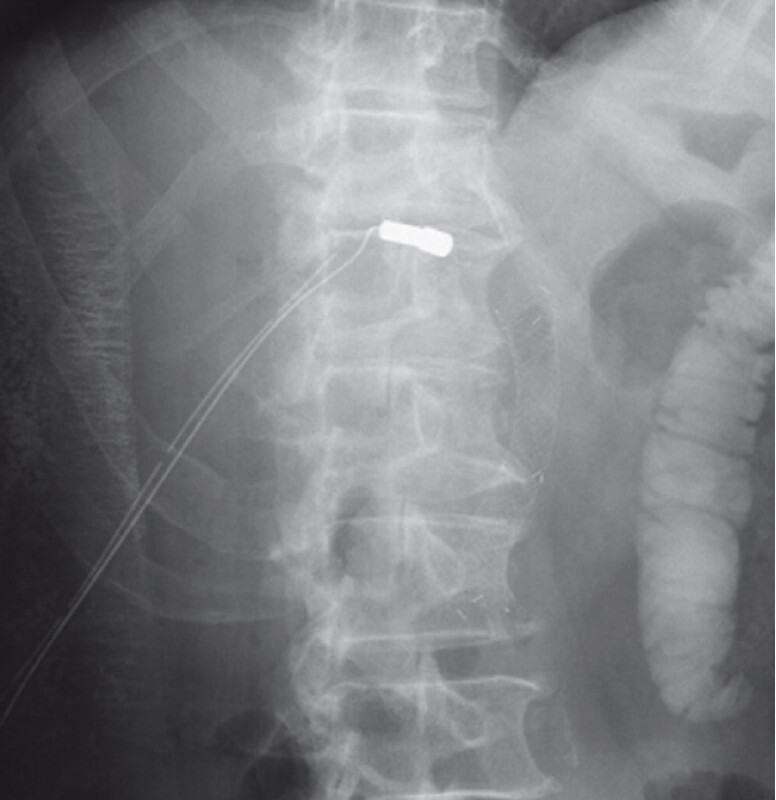
Fluoroscopic image showing the locations of the magnets.

**Fig. 4:**
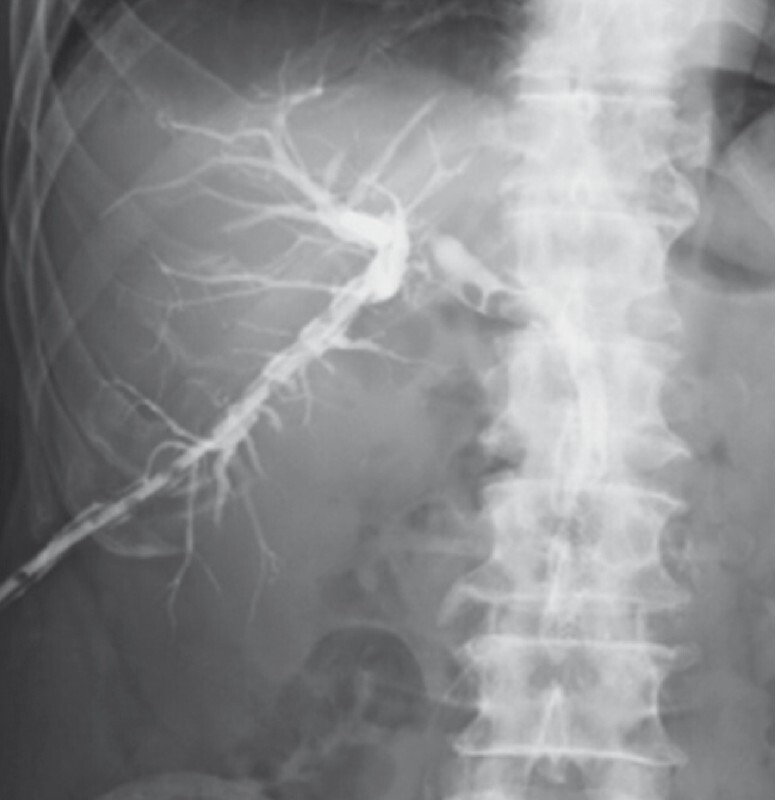
Fluoroscopic image showing recanalization following magnetic compression anastomosis.

**Fig. 5:**
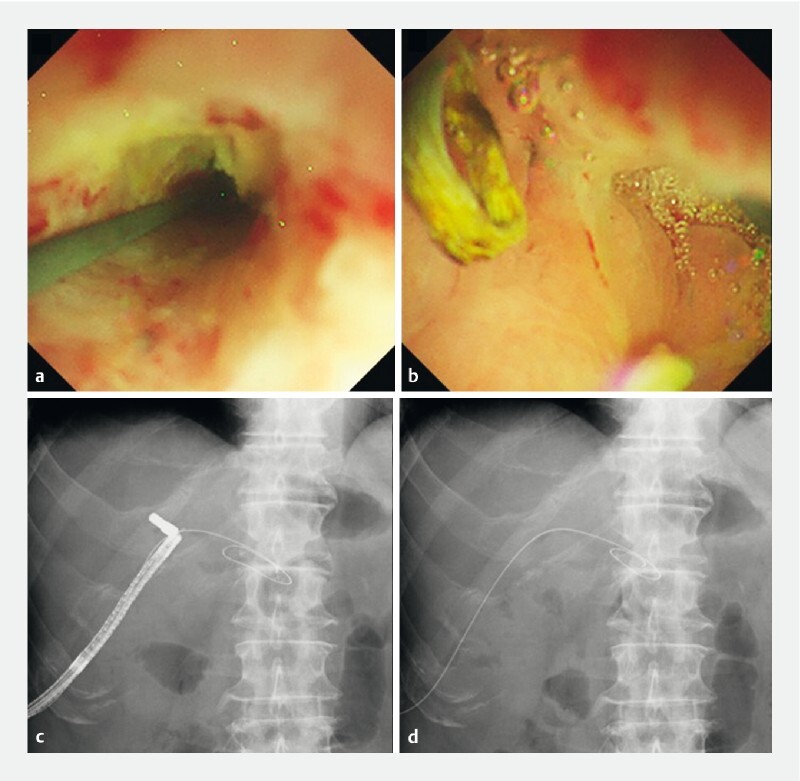
Endoscopic and fluoroscopic images showing retrieval of the magnet that migrated to the intrahepatic bile duct side. **a** Endoscopic image of the percutaneous transhepatic biliary drainage route passing through the fistula formation site. **b** Endoscopic image of thread attached to the magnet. **c** Fluoroscopic image of forceps grasping the thread attached to the magnet. **d** Fluoroscopic image of successful retrieval of the magnet.

There have been several reports of magnetic compression anastomosis 1 2 3 4 5 , but none have described retrieval of a magnet that migrated from the PTBD route. It is important to consider not only the ERCP route but also the PTBD route for retrieval of migrated magnets.

Endoscopy_UCTN_Code_CPL_1AK_2AZ

## Notes

Endoscopy E-Videos

https://eref.thieme.de/e-videos
*E-Videos* is an open access online section of the journal *Endoscopy* , reporting on interesting cases and new techniques in gastroenterological endoscopy. All papers include a high-quality video and are published with a Creative Commons CC-BY license. Endoscopy E-Videos qualify for HINARI discounts and waivers and eligibility is automatically checked during the submission process. We grant 100% waivers to articles whose corresponding authors are based in Group A countries and 50% waivers to those who are based in Group B countries as classified by Research4Life (see: https://www.research4life.org/access/eligibility/ ). This section has its own submission website at https://mc.manuscriptcentral.com/e-videos
